# Examining the causal association between moderate alcohol consumption and cardiovascular risk factors in the Taiwan Biobank: a Mendelian randomization analysis

**DOI:** 10.3389/fcvm.2024.1456777

**Published:** 2024-10-02

**Authors:** Pei-Shan Chien, Tzu-Jung Wong, An-Shun Tai, Yau-Huo Shr, Tsung Yu

**Affiliations:** ^1^ Department of Chinese Medicine, Chi Mei Medical Center, Liouying, Taiwan; ^2^ Department of Public Health, College of Medicine, National Cheng Kung University, Tainan, Taiwan; ^3^ Department of Healthcare Information and Management, School of Health and Medical Engineering, Ming Chuan University, Taipei, Taiwan; ^4^ Department of Statistics, National Cheng Kung University, Tainan, Taiwan; ^5^ Department of Agricultural Economics, National Taiwan University, Taipei, Taiwan

**Keywords:** Mendelian randomization, alcohol drinking, cardiovascular risk factors, Taiwan Biobank, causal inference

## Abstract

**Background:**

The Mendelian randomization approach uses genetic variants as instrumental variables to study the causal association between the risk factors and health outcomes of interest. We aimed to examine the relation between alcohol consumption and cardiovascular risk factors using two genetic variants as instrumental variables: alcohol dehydrogenase 1B (*ADH1B*) rs1229984 and aldehyde dehydrogenase 2 (*ALDH2*) rs671.

**Methods:**

Using data collected in the Taiwan Biobank—an ongoing, prospective, population-based cohort study—our analysis included 129,032 individuals (46,547 men and 82,485 women) with complete data on *ADH1B* rs1229984 and *ALDH2* rs671 genotypes and alcohol drinking status. We conducted instrumental variables regression analysis to examine the relationship between alcohol drinking and body mass index (BMI), systolic blood pressure (SBP), diastolic blood pressure (DBP), fasting glucose, glycated hemoglobin (HbA1c), triglycerides, high-density lipoprotein cholesterol (HDLc), and low-density lipoprotein cholesterol (LDLc).

**Results:**

In the rs1229984-instrumented analysis, alcohol drinking was only associated with higher levels of SBP in men and lower levels of DBP in women. In the rs671-instrumented analysis, alcohol drinking was associated with higher levels of BMI, SBP, DBP, fasting glucose, triglycerides, HDLc and lower levels of LDLc in men; alcohol drinking was associated with higher levels of HDLc and lower levels of SBP, HbA1c, and triglycerides in women.

**Conclusion:**

Using Mendelian randomization analysis, some of our study results among men echoed findings from the previous systematic review, suggesting that alcohol drinking may be causally associated with higher levels of BMI, SBP, DBP, fasting glucose, triglycerides, HDLc, and lower levels of LDLc. Although alcohol drinking is beneficial to a few cardiovascular risk factors, it is detrimental to many others. The assumptions that underlie the Mendelian randomization approach should also be carefully examined when interpreting findings from such studies.

## Introduction

Is moderate drinking beneficial to cardiovascular health? Several observational studies have been conducted to examine the effect of moderate alcohol consumption on the decreased risk of cardiovascular disease (1), but such studies were subject to confounding or reverse causation (2). Systematic review and meta-analysis of interventional studies that assessed the effect of alcohol on cardiovascular biomarkers have suggested that alcohol increased the levels of high-density lipoprotein cholesterol (HDLc) and adiponectin (a marker of lower risk of diabetes) (3). However, there was also evidence from interventional studies that showed that reducing alcohol intake could lower levels of blood pressure (4), suggesting the adverse effect of alcohol on blood pressure. These experimental studies regarding alcohol and health generated causal evidence but were mostly small or had assessed mainly short-term effects.

Since 2008, the Mendelian randomization approach has been used to study the effect of alcohol consumption on health (5). The Mendelian randomization approach uses genetic variants as instrumental variables to study the causal association between risk factors and health outcomes of interest (6–8). Genetic variants are allocated at conception at random and are assumed to be independent of potential confounding factors. Genetic variants also proxy for lifetime exposure and are less likely to be influenced by reverse causality. Thereby, the Mendelian randomization analysis has been widely applied to many populations in the world to assess causality and is a rapidly developing field; a systematic review of Mendelian randomization studies on alcohol consumption and cardiovascular diseases recently identified 24 studies of this kind (5).

The primary pathway of alcohol metabolism involves two enzymes: alcohol dehydrogenase (ADH) and aldehyde dehydrogenase (ALDH). ADH metabolizes alcohol to acetaldehyde, a toxic byproduct; ALDH metabolizes acetaldehyde to acetate. Accumulation of acetaldehyde in the body may cause discomfort, nausea, and facial flushing, and is associated with less alcohol consumption and lower risk of alcohol use disorders (9).

Genetic variants in the *ALDH2* (rs671) and *ADH1B* (rs1229984) are common in East Asians. Compared with other ethnic groups, East Asians, including residents in Taiwan, are more likely to carry the rs671 A allele, which decreases the rate at which acetaldehyde is metabolized to acetate, and also the rs1229984 T allele, which increases the rate at which alcohol is metabolized to acetaldehyde (10, 11) (see Supplementary Figure S1). Both genetic variants are associated with less alcohol consumption in Taiwanese populations and can serve as good genetic instruments in a Mendelian randomization analysis that examines the health effects of alcohol. However, there has been a lack of studies conducted in Taiwanese populations to address this research question.

Accordingly, we conducted a Mendelian randomization study to investigate the causal effect of alcohol consumption on cardiovascular risk factors among participants in the Taiwan Biobank. We used two genetic variants as the instrumental variables in our analysis: *ADH1B* rs1229984 and *ALDH2* rs671.

## Materials and methods

### Study population and design

The Taiwan Biobank is an ongoing, prospective, population-based cohort study that recruits individuals from more than 30 sites across Taiwan (12, 13). The study included individuals aged between 20 and 70 years who had not been diagnosed with cancer at enrollment. As of March 2024, 202,979 individuals participated in the study. We used the baseline data of 163,888 individuals that were released by the Taiwan Biobank study team in January 1st, 2023. At baseline, all individuals signed the informed consent and completed the study questionnaires, physical examinations, and blood and urine tests. Our study protocol was approved by the Research Ethics Committee at the National Taiwan University (202107HM007). Information that could identify individual participants was not available to all authors.

### Genotyping

The Taiwan Biobank study team developed two single-nucleotide polymorphism (SNP) arrays for genotyping. In 2011, the TWBv1 array was designed based on the Thermo Fisher Axiom Genome-Wide CHB Array with customized content; in 2017, the TWBv2 array was designed to enrich the content of rare coding risk alleles (13). The *ADH1B* rs1229984 and *ALDH2* rs671 polymorphism was determined using the TWBv1 or TWBv2 arrays. The genotype frequencies of both SNPs did not deviate from the Hardy-Weinberg equilibrium (*P* = 0.304 for rs1229984 and *P* = 0.941 for rs671).

### Alcohol consumption and covariates

Alcohol drinking was assessed using questionnaires and was categorized into never or seldom drinkers, former drinkers and current drinkers. In the Mendelian randomization analysis, we created a binary variable of alcohol drinking that compared drinkers (current or former) to non-drinkers (never or seldom). Other covariates included education (elementary school, junior high school, senior high school, college or university or graduate school), marital status (single, married, separate/divorced or widowed), smoking (never or seldom, former or current), and regular exercise (yes or no). The history of alcohol or drug abuse, cardiovascular disease, hyperlipidemia, hypertension, and diabetes were assessed by self-reports of participants. We classified all drinkers as having moderate alcohol consumption based on our previous research (unpublished data), which indicated that the majority of people in Taiwan are either occasional or moderate drinkers. Additionally, we excluded individuals with a history of alcohol abuse.

### Cardiovascular risk factors

Measurements regarding cardiovascular risk factors included body mass index (BMI), systolic blood pressure (SBP), diastolic blood pressure (DBP), fasting glucose, glycated hemoglobin (HbA1c), triglycerides, high-density lipoprotein cholesterol (HDLc), and low-density lipoprotein cholesterol (LDLc). Weight in kilograms and height in meters were used to calculate BMI. Measurement of SBP and DBP was taken three times at short intervals using a sphygmomanometer and we calculated the mean of these values. Blood samples were analyzed using an antoanalyzer (Roche Diagnostics GmbH, D-68298 Mannheim COBAS Integra 400) to obtain data on fasting glucose, HbA1c, triglycerides, HDLc, and LDLc (12).

### Statistical analysis

We conducted statistical analysis using STATA, version 15 (Stata Corporation, College Station, Texas, USA). Our analysis included 129,032 individuals (46,547 men and 82,485 women) with complete data on rs1229984 and rs671 genotypes and alcohol drinking. Given that in East Asian countries drinking behavior differs greatly between men and women, we conducted analysis separately for men and women (14).

*ADH1B* rs1229984 can be categorized into TT, CT, and CC allele groups, with the CC group more likely to drink alcohol. *ALDH2* rs671 can be categorized into AA, GA, and GG allele groups, with the GG group more likely to drink alcohol.

Distributions of fasting glucose, HbA1c, and triglycerides were found skewed, so we log transformed the data to improve normality. We used linear regression to examine the associations of *ADH1B* rs1229984 and *ALDH2* rs671 genotypes with cardiovascular risk factors. We used chi-squared tests to examine the associations of *ADH1B* rs1229984 and *ALDH2* rs671 genotypes with the exposure (alcohol drinking) and potential confounding factors. At first, we performed multivariable linear regression to examine the relationship between alcohol drinking and cardiovascular risk factors, with adjustment for age, education, marital status, smoking, and exercise habits. Then, we conducted instrumental variables regression analysis (*ivregress* command in STATA) to examine the same relationship, using *ADH1B* rs1229984 and *ALDH2* rs671 genotypes as instruments. This method allowed us to estimate the effect of alcohol drinking on cardiovascular risk factors, with results presented as regression coefficients along with 95% confidence intervals. The analysis determines this effect by dividing the relationship between the genetic instrument and the cardiovascular risk factor by the relationship between genetic instrument and alcohol drinking, thus utilizing the connections among genotypes, alcohol drinking, and cardiovascular risk factors. An F statistic was calculated to evaluate the strength of the instrument in the first stage regression, with values smaller than 10 considered to be of weak instruments. We calculated the Durbin-Wu-Hausman statistic to compare the effect estimates obtained from linear regression with that from the instrumental variables regression (15).

## Results

Table 1 shows the characteristics of study participants, including 46,547 men and 82,485 women. In men, 67.2% graduated from college, university, or graduate school; the corresponding proportion for women was 53.7%. More men were married than women, while more women were separated, divorced, or widowed than men. In men, 13.1% were classified as current drinkers and 5.6% were former drinkers; for women, the corresponding proportions were 1.9% and 0.9%. In men, 20.7% were classified as current smokers and 23.4% were former smokers; the corresponding proportions were 3.0% and 2.6% for women. In general, men had worse cardiovascular disease risk factor profiles than women.

**Table 1:** Characteristics of study participants according to sex.

Characteristics	Men (*n* = 46,547)	Women (*n* = 82,485)
	Mean (SD) or *n* (%)	Mean (SD) or *n* (%)
Age in years, *n* (%)		
<40	11,358 (24.4)	18,388 (22.3)
40–49	11,226 (24.1)	20,151 (24.4)
50–59	12,249 (26.3)	25,886 (31.4)
≥60	11,714 (25.2)	18,060 (21.9)
Education, *n* (%)		
Elementary school	1,331 (2.9)	5,184 (6.3)
Junior high school	2,553 (5.5)	6,828 (8.3)
Senior high school	11,355 (24.4)	26,130 (31.7)
College or university	24,018 (51.6)	37,264 (45.2)
Graduate school	7,281 (15.7)	7,054 (8.6)
Marital status, *n* (%)		
Single	6,546 (14.1)	11,566 (14.0)
Married	36,819 (79.1)	57,425 (69.7)
Separate/divorced	2,684 (5.8)	8,232 (10.0)
Widowed	486 (1.0)	5,208 (6.3)
Alcohol drinking, *n* (%)		
Never or seldom	37,838 (81.3)	80,150 (97.2)
Former	2,624 (5.6)	742 (0.9)
Current	6,085 (13.1)	1,593 (1.9)
Smoking, *n* (%)		
Never or seldom	26,050 (56.0)	77,803 (94.3)
Former	10,874 (23.4)	2,156 (2.6)
Current	9,615 (20.7)	2,508 (3.0)
Regular exercise, *n* (%)	19,442 (41.8)	32,152 (39.0)
Alcohol or drug abuse, *n* (%)	43 (0.1)	7 (0.0)
History of CVD or stroke, *n* (%)	4,716 (10.1)	8,481 (10.3)
High blood lipids, *n* (%)	4,369 (9.4)	5,301 (6.4)
High blood pressure, *n* (%)	7,802 (16.8)	7,897 (9.6)
Diabetes mellitus, *n* (%)	3,173 (6.8)	3,486 (4.2)
BMI in kg/m^2^, mean (SD)	25.4 (3.6)	23.6 (3.8)
SBP in mmHg, mean (SD)	125.3 (16.4)	116.2 (17.8)
DBP in mmHg, mean (SD)	78.0 (10.6)	70.7 (10.3)
Fasting glucose in mg/dl, mean (SD)	99.3 (23.2)	94.0 (18.7)
HbA1c in%, mean (SD)	5.8 (0.9)	5.7 (0.7)
Triglycerides in mg/dl, mean (SD)	138.9 (118.9)	103.3 (74.7)
HDLc in mg/dl, mean (SD)	48.0 (11.1)	58.3 (13.3)
LDLc in mg/dl, mean (SD)	121.9 (31.6)	120.5 (31.9)

BMI, body mass index; CVD, cardiovascular disease; DBP, diastolic blood pressure; HbA1c, glycated hemoglobin; HDLc, high density lipoprotein cholesterol; LDLc, low density lipoprotein cholesterol; SBP, systolic blood pressure; SD, standard deviation.

As Table 2 shows, the *ADH1B* rs1229984 genotype was associated with alcohol drinking in men and women and it was not associated with other potential confounding factors. As Table 3 shows, the *ALDH2* rs671 genotype was associated with alcohol drinking and it was related to some potential confounding factors in men and in women, although the differences in the distribution of confounding factors among different rs671 genotypes were small. The F statistic that evaluated the strength of the instruments was against weak instrument bias. In men, the F values were 46.1 for *ADH1B* rs1229984 and 2418.4 for *ALDH2* rs671. In women, the F values were 40.0 for *ADH1B* rs1229984 and 667.0 for *ALDH2* rs671.

**Table 2:** Associations of rs1229984 genotype with alcohol drinking and potential confounders in men and women.

Characteristics	Number of C alleles			
	0	1	2	*P*
Men (*n* = 46,547)				
Number of participants	25,247	18,114	3,186	
Alcohol drinking,%				<0.001
Never or seldom	81.9	81.3	76.2	
Former	5.7	5.6	5.8	
Current	12.4	13.1	17.9	
Age in years, %				0.845
<40	24.4	24.2	25.2	
40–49	24.1	24.1	24.2	
50–59	26.5	26.2	25.6	
≥60	25.0	25.4	25.0	
Education, %				0.365
Elementary school	2.9	2.8	2.8	
Junior high school	5.6	5.5	4.9	
Senior high school	24.7	23.9	25.2	
College or university	51.3	52.1	51.5	
Graduate school	15.5	15.8	15.7	
Marital status, %				0.320
Single	13.7	14.4	14.8	
Married	79.4	79.0	78.1	
Separate/divorced	5.9	5.6	6.0	
Widowed	1.0	1.0	1.1	
Smoking, %				0.134
Never or seldom	55.8	56.5	54.5	
Former	23.3	23.3	23.9	
Current	20.9	20.2	21.6	
Regular exercise, %	41.8	41.9	41.3	0.798
Women (*n* = 82,485)				
Number of participants	44,644	31,881	5,960	
Alcohol drinking, %				<0.001
Never or seldom	97.4	97.2	95.7	
Former	0.9	0.9	0.9	
Current	1.7	1.9	3.4	
Age in years, %				0.647
<40	22.3	22.4	21.9	
40–49	24.6	24.3	24.0	
50–59	31.3	31.3	32.5	
≥60	21.9	21.9	21.6	
Education, %				0.720
Elementary school	6.3	6.3	6.3	
Junior high school	8.5	8.1	7.8	
Senior high school	31.6	31.8	32.1	
College or university	45.1	45.4	45.2	
Graduate school	8.6	8.5	8.6	
Marital status, %				0.976
Single	14.0	14.1	14.2	
Married	69.8	69.5	69.3	
Separate/divorced	10.0	10.0	10.1	
Widowed	6.3	6.4	6.4	
Smoking, %				0.952
Never or seldom	94.4	94.4	94.3	
Former	2.6	2.6	2.5	
Current	3.0	3.0	3.2	
Regular exercise, %	39.0	38.9	39.3	0.852

**Table 3:** Associations of rs671 genotype with alcohol drinking and potential confounders in men and women.

Characteristics	Number of G alleles			
	0	1	2	*P*
Men (*n* = 46,547)				
Number of participants	3,839	18,975	23,733	
Alcohol drinking, *n* (%)				<0.001
Never or seldom	98.4	87.5	73.6	
Former	1.0	4.9	7.0	
Current	0.6	7.6	19.5	
Age in years, *n* (%)				0.030
<40	24.6	24.0	24.7	
40–49	25.2	23.9	24.1	
50–59	25.4	26.2	26.6	
≥60	24.9	25.9	24.6	
Education, *n* (%)				0.005
Elementary school	3.5	3.0	2.6	
Junior high school	5.8	5.6	5.3	
Senior high school	24.7	24.8	24.0	
College or university	51.2	51.3	52.0	
Graduate school	14.9	15.3	16.0	
Marital status, *n* (%)				0.070
Single	14.4	13.9	14.2	
Married	78.6	78.9	79.4	
Separate/divorced	5.8	6.1	5.5	
Widowed	1.3	1.1	0.9	
Smoking, *n* (%)				0.002
Never or seldom	59.1	55.9	55.6	
Former	21.4	23.4	23.6	
Current	19.5	20.7	20.8	
Regular exercise, *n* (%)	38.4	41.9	42.3	<0.001
Women (*n* = 82,485)				
Number of participants	6,506	33,298	42,681	
Alcohol drinking, *n* (%)				<0.001
Never or seldom	99.8	98.5	95.8	
Former	0.2	0.7	1.2	
Current	0.0	0.9	3.1	
Age in years, *n* (%)				0.286
<40	23.3	22.4	22.0	
40–49	24.5	24.5	24.4	
50–59	30.6	31.4	31.5	
≥60	21.6	21.7	22.1	
Education, *n* (%)				0.011
Elementary school	6.8	6.5	6.0	
Junior high school	8.4	8.4	8.2	
Senior high school	32.2	31.7	31.6	
College or university	44.9	45.0	45.4	
Graduate school	7.8	8.5	8.7	
Marital status, *n* (%)				<0.001
Single	15.8	13.9	13.9	
Married	68.4	70.0	69.6	
Separate/divorced	9.9	9.9	10.1	
Widowed	5.9	6.2	6.5	
Smoking, *n* (%)				0.004
Never or seldom	94.6	94.6	94.1	
Former	2.3	2.4	2.8	
Current	3.1	3.0	3.0	
Regular exercise, *n* (%)	37.1	38.6	39.6	<0.001

In the supplementary materials, Supplementary Tables S1 and S2 show the associations of rs1229984 and rs671 with cardiovascular disease risk factors, respectively. In men, a larger number of *ADH1B* rs1229984 C alleles (more likely to be drinkers) was associated with higher levels of SBP. In women, however, a larger number of *ADH1B* rs1229984 C alleles was associated with lower levels of DBP. In men, a larger number of *ALDH2* rs671 G alleles (more likely to be drinkers) was associated with higher levels of BMI, SBP, DBP, triglycerides, HDLc, and lower levels of LDLc. In women, a larger number of *ALDH2* rs671 G alleles was associated with higher levels of HDLc, and lower levels of SBP, HbA1c and triglycerides.

Supplementary Tables S3 and S4 show the associations of rs1229984 and rs671 with cardiovascular disease risk factors with adjustment for alcohol drinking. Some of these coefficients were attenuated after adjustment but remained statistically significant.

Table 4 shows the associations of alcohol drinking (current or former drinkers vs. never or seldom drinkers) with cardiovascular disease risk factors in men and women, using both standard multivariable linear regression models and instrumental variables regression models. In men, standard multivariable linear regression analysis suggested that alcohol drinking was associated with higher levels of BMI, SBP, DBP, fasting glucose, triglycerides, HDLc, and lower levels of LDLc. In rs1229984-instrumented analysis, alcohol drinking was only associated with SBP. In rs671-instrumented analysis, the results were similar to the standard multivariable linear regression analysis, but the coefficients were larger, indicating stronger associations.

**Table 4:** Confounder adjusted multivariable and instrumental variable associations of alcohol drinking with cardiovascular disease risk factors.

	Mean difference of each outcome comparing current or former drinkers to never or seldom drinkers															
	BMI		SBP		DBP		Log transformed fasting glucose		Log transformed HbA1c		Log transformed triglycerides		HDLc		LDLc	
	*β*	*P*	β	*P*	β	*P*	β	*P*	β	*P*	β	*P*	β	*P*	β	*P*
Men (*n* = 46,547)																
Multivariable analysis^a^																
	**0**.**252**	**<0**.**001**	**2**.**933**	**<0**.**001**	**2**.**435**	**<0**.**001**	**0**.**016**	**<0**.**001**	−0.001	0.626	**0**.**083**	**<0**.**001**	**2**.**458**	**<0**.**001**	**−4**.**352**	**<0**.**001**
Instrumental variable analysis^a^																
rs1229984	1.382	0.330	**17**.**642**	**0**.**007**	5.203	0.215	−0.075	0.253	−0.090	0.071	0.029	0.897	4.194	0.333	−0.127	0.992
*P* for difference^b^	0.422		**0**.**018**		0.507		0.155		0.063		0.808		0.688		0.735	
rs671	**1**.**064**	**<0**.**001**	**5**.**833**	**<0**.**001**	**5**.**284**	**<0**.**001**	**0**.**023**	**0**.**011**	−0.006	0.417	**0**.**233**	**<0**.**001**	**6**.**161**	**<0**.**001**	**−6**.**762**	**<0**.**001**
*P* for difference^b^	**<0**.**001**		**<0**.**001**		**<0**.**001**		0.443		0.471		**<0**.**001**		**<0**.**001**		0.162	
Women (*n* = 82,485)																
Multivariable analysis																
	**−0**.**194**	**0**.**016**	**0**.**693**	**0**.**043**	**1**.**149**	**<0**.**001**	0.001	0.777	**−0**.**014**	**<0**.**001**	0.019	0.086	**4**.**116**	**<0**.**001**	**−2**.**748**	**<0**.**001**
Instrumental variable analysis																
rs1229984	3.060	0.407	−32.027	0.050	**−23**.**281**	**0**.**027**	−0.256	0.070	−0.187	0.067	−0.871	0.091	5.420	0.674	5.451	0.858
*P* for difference	0.373		**0**.**035**		**0**.**013**		0.058		0.078		0.072		0.919		0.788	
rs671	0.766	0.394	**−15**.**550**	**<0**.**001**	−1.522	0.529	−0.050	0.137	**−0**.**056**	**0**.**021**	**−0**.**403**	**0**.**001**	**10**.**345**	**0**.**001**	−11.388	0.128
*P* for difference	0.283		**<0**.**001**		0.267		0.128		0.083		**<0**.**001**		**0**.**048**		0.246	

BMI, body mass index; DBP, diastolic blood pressure; HbA1c, glycated hemoglobin; HDLc, high density lipoprotein cholesterol; LDLc, low density lipoprotein cholesterol; SBP, systolic blood pressure.

^a^ Adjusted for age, education, marital status, smoking and exercise habits.

^b^ Durbin-Wu-Hausman statistic.

*P*-values <0.05 are highlighted in bold.

In women, standard multivariable linear regression analysis suggested that alcohol drinking was associated with higher levels of SBP, DBP, HDLc, and lower levels of BMI, HbA1c, and LDLc (Table 4). Results of instrumental variables regression analysis in women were less consistent with that of multivariable linear regression analysis. In rs1229984-instrumented analysis, alcohol drinking was associated with lower levels of DBP. In rs671-instrumented analysis, alcohol drinking was associated with higher levels of HDLc and lower levels of SBP, HbA1c, and triglycerides. We also conducted sensitivity analysis where we excluded participants with a history of alcohol abuse, cardiovascular disease or stroke, and the results were similar to our primary analysis (see Supplementary Table S3). For clarity, all study results are summarized and presented in Supplementary Table S6, with data stratified by sex.

## Discussion

Among our study participants, more men (18.7%) than women (2.8%) were former drinkers or current drinkers, since in East Asian culture social pressure precludes alcohol drinking in women (16), while in Western countries this alcohol drinking culture does not apply. Besides, the *ALDH2* rs671 genotype was more associated with alcohol drinking than the *ADH1B* rs1229984 genotype. We therefore used the results of rs671-instrumented analysis in men as the primary finding.

Alcohol drinking may cause higher levels of BMI, SBP, DBP, fasting glucose, triglycerides, HDLc, and lower levels of LDLc. The associations in instrumental variables regression analysis were consistent with that in standard multivariable regression analysis, but the effect sizes were larger.

To our knowledge, our study was one of the first studies that used the Mendelian randomization approach to examine the casual association between alcohol consumption and cardiovascular risk factors in Taiwan, and thereby contributed new data to the current literature.

The results of rs1229984-instrumented analysis suggested that alcohol drinking may cause higher levels of SBP only in men and lower levels of DBP in women. The F statistics that evaluated the strength of the instruments were 46.1 for *ADH1B* rs1229984 in men and 40.0 for *ADH1B* rs1229984 in women, respectively, which was against weak instrument. However, the rs1229984 genotype may not be a good instrument in our study population, as there was a lack of association between alcohol drinking and cardiovascular health identified in the rs1229984-instrumented analysis. This may be the reason that previous studies conducted in Asian countries mostly used *ALDH2* rs671 genotype as the genetic instrument, and *ADH1B* rs1229984 was more used in populations of European ancestry (5). Caucasians do not have the *ALDH2* rs671 polymorphism, so studies involving this population would need to focus on *ADH* polymorphisms, which show weaker associations with alcohol consumption in Asians.

A recent systematic review of Mendelian randomization studies regarding alcohol consumption in relation to cardiovascular diseases and mortality included 24 studies (5). For cardiovascular disease risk factors in general, those authors found that alcohol drinking was detrimental to blood pressure, blood glucose, triglycerides, and BMI while it was protective of HDLc and LDLc. Interestingly, our primary findings also accorded well with the findings reported in that systematic review. Hence, it is very likely that alcohol drinking is causally associated with higher levels of BMI, SBP, DBP, fasting glucose, triglycerides, HDLc, and lower levels of LDLc. However, we still need to be cautious about the limitations pertinent to Mendelian randomization studies when we interpret these results.

The Mendelian randomization approach uses genetic variants as instrumental variables to strengthen causal inference. To ensure the validity of a genetic instrument, we can check whether it fulfills three key assumptions: (1) The genetic variant is robustly associated with the exposure of interest; (2) The genetic variant is independent of the confounding factors that confound the association between the exposure and outcome; (3) The genetic variant does not affect the outcome, except via its association with the exposure.

For the first assumption, we found a strong association between the genetic variants (*ADH1B* rs1229984 and *ALDH2* rs671) and the exposure (alcohol drinking). However, this association was much smaller in women than in men, which reflects that in Taiwanese culture women tend to abstain from alcohol drinking.

For the second assumption—because at conception genetic variants are randomly allocated—we can therefore assume that genetic variants are independent of factors that may confound the association between the exposure and outcome. In the present study, we found that the *ADH1B* rs1229984 genotype was not related to potential confounding factors (see Table 2). Conversely, we found that, although the distribution of potential confounding factors were similar according to different *ALDH2* rs671 genotypes, there seemed to be weak associations of *ALDH2* rs671 genotypes with confounding factors. For instance, our study participants who carried more rs671 G alleles (more likely to drink alcohol) tended to be slightly more educated and physically active (see Table 3).

For the third assumption, the *ADH1B* rs1229984 and *ALDH2* rs671 genotypes should influence the outcomes only via their association with the exposure of interest. We checked this assumption by examining whether the associations of genetic variants and outcomes still held after we adjusted for alcohol drinking (Supplementary Tables S3 and S4).

In the present study, we found that the associations of *ADH1B* rs1229984 and *ALDH2* rs671 genotypes with outcomes were not much attenuated after adjustment for alcohol drinking. This may suggest violation of the exclusion-restriction assumption and that genetic variants have horizontal pleiotropic effects.

### Study limitations and violation of assumptions

Some important limitations of the present study might have led to the violation of assumptions relevant to the Mendelian randomization approach. First, our assessment of the exposure (alcohol drinking) was not ideal. We assessed alcohol drinking using the question, “Do you now drink alcohol (at least 150 ml per week for 6 months)?”, and we categorized alcohol drinking status into “never or seldom,” “former,” and “current.” We did not ask detailed questions regarding the type, intensity, and frequency of alcohol use, so we could not define our exposure using continuous measures. Hence, we might have misclassified alcohol drinking status in our study participants, leading to information bias.

Second, we were surprised to find that there were weak associations of *ALDH2* rs671 genotypes with potential confounding factors in our study. Given that genetic variants are randomly allocated at conception, they should not be associated with confounders of the exposure-outcome association. We think that selection bias might have played a role in our data (17). For example, because the Taiwan Biobank was a prospective population-based cohort study that recruited adults in Taiwan (20–70 years of age) who had not been diagnosed with cancer, they tended to be healthier than the general population. It is likely that individuals who carried fewer *ALDH2* rs671 G alleles (less likely to drink alcohol) or individuals who had higher educational levels were more likely to participate in the study. By conditioning on the study participation, individuals who carried fewer *ALDH2* rs671 G alleles may have been inversely associated with having higher educational levels, which reflected an artifact of selection bias.

Finally, the violation of the exclusion-restriction assumption suggested that genetic variants may have horizontal pleiotropic effects. It has been reported that *ALDH2* dysfunction is associated with a variety of human diseases, including cardiovascular diseases, diabetes mellitus, neurodegenerative diseases, alcohol-induced pathophysiology, and upper aerodigestive track cancers. Recent evidence also pointed out that *ALDH2* dysfunction is relevant to Fanconi anemia, pain, osteoporosis, and human aging (18). That the *ALDH2* rs671 polymorphism is associated with a good amount of diseases suggested that there may be several pathways from *ALDH2* rs671 genotypes to disease outcomes that are not mediated through alcohol drinking. These violations of assumptions may result in discrepancies between our findings and those of previous research.

Other limitations include the lack of analysis on cardiovascular endpoints. Since our study was cross-sectional and relied on self-reported data, the cardiovascular endpoints data may not be as reliable or valid as those obtained from medical records. Our study's findings may not be generalizable to other populations, as differences in sample sizes, instruments, ethnic groups, and laboratory measures across studies could affect the results.

## Conclusions

Regardless of these limitations, our study echoes the findings from the systematic review considering the causal association between alcohol drinking and higher levels of BMI, SBP, DBP, fasting glucose, triglycerides, HDLc, and lower levels of LDLc. Although alcohol drinking may be beneficial to a few cardiovascular risk factors, it is detrimental to many aspects of cardiovascular health. Moderate drinking may not be advisable as a public health intervention for improving cardiovascular health. The assumptions that underlie the Mendelian randomization approach should also be carefully examined when we attempt to conduct such studies in the future. We hope that future Mendelian randomization studies will utilize more advanced methods and larger study sample sizes to help resolve the debate on the health effects of moderate drinking.

## Data Availability

The data analyzed in this study is subject to the following licenses/restrictions: All data of the Taiwan Biobank participants are available upon application for research purposes. A detailed description of Taiwan Biobank data availability and the application process can be found at https://www.biobank.org.tw/. Requests to access these datasets should be directed to Tsung Yu, tsung.yu.ncku@gmail.com.
