# Supplementary material for: Examining the causal association between moderate alcohol consumption and cardiovascular risk factors in the Taiwan Biobank: a Mendelian randomization analysis
Source: Front Cardiovasc Med. 2024 Oct 2;11:1456777. doi: 10.3389/fcvm.2024.1456777 (PMC11480056; doi:10.3389/fcvm.2024.1456777)
Supplement: Supplementary file 1 [file Datasheet1.docx]

**Examining the Causal Association Between Alcohol Consumption**

**and Cardiovascular Risk Factors in the Taiwan Biobank:**

**a Mendelian Randomization Analysis**

**Pei-Shan Chien, Tzu-Jung Wong, An-Shun Tai, Yau-Huo Shr, Tsung Yu**

**Supplementary Materials**


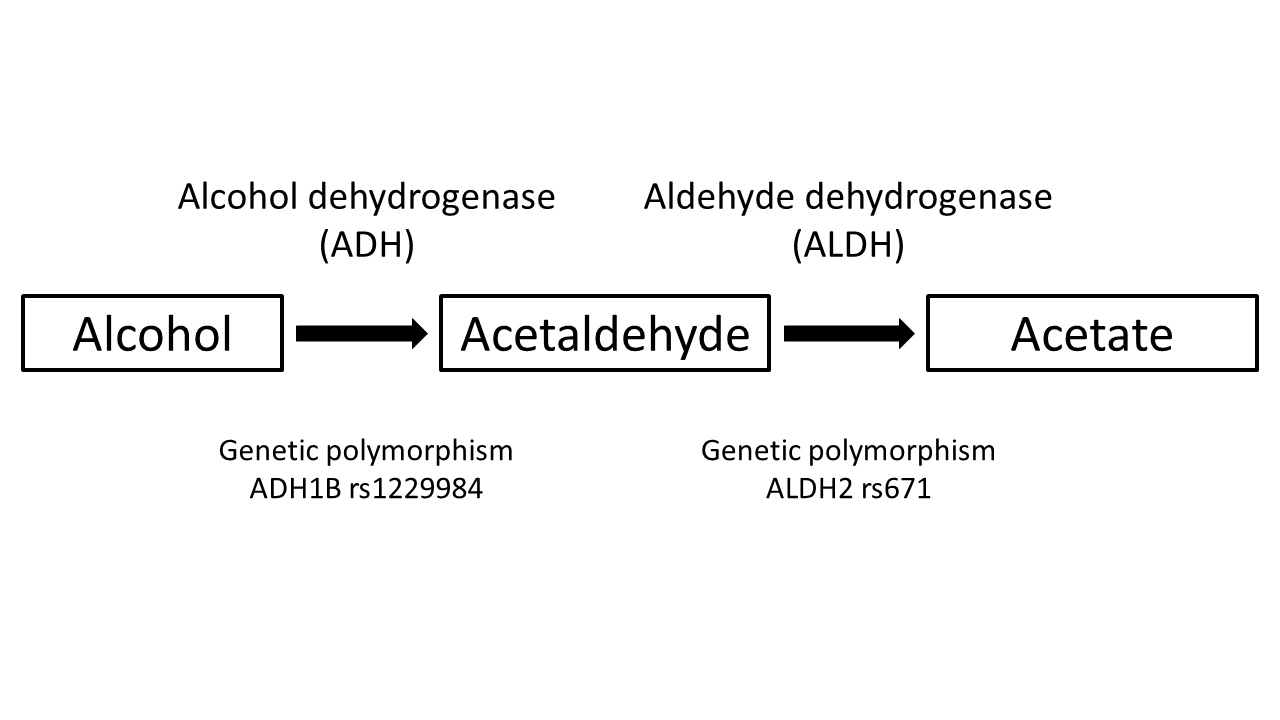


**Figure S1. Major pathway of alcohol metabolism.**

**Table S1. Associations of rs1229984 genotype with outcomes in men and women**

| Predictor | BMI | | SBP | | DBP | | Log transformed fasting glucose | | Log transformed HbA1c | | Log transformed triglycerides | | HDLc | | LDLc | |
| --- | --- | --- | --- | --- | --- | --- | --- | --- | --- | --- | --- | --- | --- | --- | --- | --- |
|  | **β** | ***P*** | **β** | ***P*** | **β** | ***P*** | **β** | ***P*** | **β** | ***P*** | **β** | ***P*** | **β** | ***P*** | **β** | ***P*** |
| Men (n = 46 547)  Number of C alleles | | | | | | | | | | | | | | | | |
| 0 | *Ref* |  | *Ref* |  | *Ref* |  | *Ref* |  | *Ref* |  | *Ref* |  | *Ref* |  | *Ref* |  |
| 1 | 0.002 | 0.952 | 0.066 | 0.681 | -0.112 | 0.278 | -0.001 | 0.504 | -0.000 | 0.789 | 0.007 | 0.236 | 0.034 | 0.751 | -0.420 | 0.172 |
| 2 | 0.081 | 0.230 | **1.184** | **<0.001** | **0.567** | **0.005** | -0.003 | 0.280 | **-0.006** | **0.014** | -0.014 | 0.208 | 0.264 | 0.206 | 0.736 | 0.216 |
| Per-allele increase | 0.022 | 0.408 | **0.341** | **0.006** | 0.094 | 0.235 | -0.001 | 0.259 | -0.002 | 0.073 | -0.000 | 0.927 | 0.085 | 0.303 | -0.009 | 0.971 |
| Women (n =82 485)  Number of C alleles | | | | | | | | | | | | | | | | |
| 0 | *Ref* |  | *Ref* |  | *Ref* |  | *Ref* |  | *Ref* |  | *Ref* |  | *Ref* |  | *Ref* |  |
| 1 | 0.025 | 0.371 | -0.180 | 0.168 | -0.110 | 0.146 | -0.001 | 0.232 | -0.001 | 0.413 | -0.003 | 0.400 | -0.080 | 0.412 | 0.205 | 0.381 |
| 2 | 0.018 | 0.724 | -0.324 | 0.188 | **-0.286** | **0.044** | -0.003 | 0.132 | -0.003 | 0.064 | -0.012 | 0.097 | 0.276 | 0.131 | -0.128 | 0.771 |
| Per-allele increase | 0.016 | 0.435 | -0.170 | 0.085 | **-0.128** | **0.025** | -0.001 | 0.083 | -0.001 | 0.082 | -0.005 | 0.104 | 0.038 | 0.609 | 0.060 | 0.736 |

**Abbreviations:**

BMI = body mass index; DBP = diastolic blood pressure; HbA1c = glycated hemoglobin; HDLc = high density lipoprotein cholesterol; LDLc = low density lipoprotein cholesterol; SBP = systolic blood pressure.

**Table S2. Associations of rs671 genotype with outcomes in men and women**

| Predictor | BMI | | SBP | | DBP | | Log transformed fasting glucose | | Log transformed HbA1c | | Log transformed triglycerides | | HDLc | | LDLc | |
| --- | --- | --- | --- | --- | --- | --- | --- | --- | --- | --- | --- | --- | --- | --- | --- | --- |
|  | **β** | ***P*** | **β** | ***P*** | **β** | ***P*** | **β** | ***P*** | **β** | ***P*** | **β** | ***P*** | **β** | ***P*** | **β** | ***P*** |
| Men (n=46547)  Number of G alleles | | | | | | | | | | | | | | | | |
| 0 | *Ref* |  | *Ref* |  | *Ref* |  | *Ref* |  | *Ref* |  | *Ref* |  | *Ref* |  | *Ref* |  |
| 1 | **0.144** | **0.023** | **0.892** | **0.002** | **0.596** | **0.002** | 0.001 | 0.638 | 0.001 | 0.605 | -0.002 | 0.812 | **0.639** | **0.001** | **-1.183** | **0.034** |
| 2 | **0.282** | **<0.001** | **1.496** | **<0.001** | **1.290** | **<0.001** | 0.004 | 0.157 | -0.001 | 0.621 | **0.040** | **<0.001** | **1.533** | **<0.001** | **-1.915** | **<0.001** |
| Per-allele increase | **0.140** | **<0.001** | **0.687** | **<0.001** | **0.666** | **<0.001** | 0.002 | 0.053 | -0.001 | 0.166 | **0.030** | **<0.001** | **0.821** | **<0.001** | **-0.861** | **<0.001** |
| Women (n=82485)  Number of G alleles | | | | | | | | | | | | | | | | |
| 0 | *Ref* |  | *Ref* |  | *Ref* |  | *Ref* |  | *Ref* |  | *Ref* |  | *Ref* |  | *Ref* |  |
| 1 | -0.042 | 0.412 | **-0.499** | **0.039** | -0.150 | 0.283 | -0.004 | 0.059 | **-0.003** | **0.043** | **-0.015** | **0.036** | **0.570** | **0.002** | -0.106 | 0.807 |
| 2 | -0.023 | 0.643 | **-0.774** | **0.001** | -0.149 | 0.278 | **-0.004** | **0.049** | **-0.004** | **0.013** | **-0.022** | **0.002** | **0.742** | **<0.001** | -0.351 | 0.408 |
| Per-allele increase | 0.002 | 0.936 | **-0.338** | **<0.001** | -0.041 | 0.465 | -0.001 | 0.163 | **-0.001** | **0.032** | **-0.009** | **0.001** | **0.284** | **<0.001** | -0.206 | 0.238 |

**Abbreviations:**

BMI = body mass index; DBP = diastolic blood pressure; HbA1c = glycated hemoglobin; HDLc = high density lipoprotein cholesterol; LDLc = low density lipoprotein cholesterol; SBP = systolic blood pressure.

**Table S3. Associations of rs1229984 genotype with outcomes in men and women, adjusted for alcohol drinking**

| Predictor | BMI | | SBP | | DBP | | Log transformed fasting glucose | | Log transformed HbA1c | | Log transformed triglycerides | | HDLc | | LDLc | |
| --- | --- | --- | --- | --- | --- | --- | --- | --- | --- | --- | --- | --- | --- | --- | --- | --- |
|  | **β** | ***P*** | **β** | ***P*** | **β** | ***P*** | **β** | ***P*** | **β** | ***P*** | **β** | ***P*** | **β** | ***P*** | **β** | ***P*** |
| Men (n=46547)  Number of C alleles | | | | | | | | | | | | | | | | |
| 0 | *Ref* |  | *Ref* |  | *Ref* |  | *Ref* |  | *Ref* |  | *Ref* |  | *Ref* |  | *Ref* |  |
| 1 | -0.000 | 0.994 | 0.044 | 0.781 | -0.128 | 0.214 | -0.001 | 0.436 | -0.000 | 0.751 | 0.006 | 0.302 | 0.025 | 0.815 | -0.393 | 0.201 |
| 2 | 0.059 | 0.381 | **0.986** | **0.001** | **0.422** | **0.034** | -0.005 | 0.109 | **-0.006** | **0.007** | **-0.022** | **0.045** | 0.180 | 0.387 | 0.984 | 0.098 |
| Per-allele increase | 0.015 | 0.567 | **0.279** | **0.023** | 0.049 | 0.536 | -0.002 | 0.124 | **-0.002** | **0.048** | -0.003 | 0.498 | 0.059 | 0.475 | 0.069 | 0.770 |
| Women (n=82485)  Number of C alleles | | | | | | | | | | | | | | | | |
| 0 | *Ref* |  | *Ref* |  | *Ref* |  | *Ref* |  | *Ref* |  | *Ref* |  | *Ref* |  | *Ref* |  |
| 1 | 0.025 | 0.370 | -0.179 | 0.171 | -0.112 | 0.140 | -0.001 | 0.232 | -0.001 | 0.439 | -0.003 | 0.390 | -0.087 | 0.368 | 0.211 | 0.367 |
| 2 | 0.019 | 0.719 | -0.313 | 0.203 | **-0.301** | **0.034** | -0.003 | 0.133 | -0.002 | 0.096 | -0.013 | 0.082 | 0.217 | 0.236 | -0.079 | 0.858 |
| Per-allele increase | 0.017 | 0.432 | -0.167 | 0.092 | **-0.132** | **0.020** | -0.001 | 0.083 | -0.001 | 0.113 | -0.005 | 0.091 | 0.018 | 0.805 | 0.076 | 0.668 |

**Abbreviations:**

BMI = body mass index; DBP = diastolic blood pressure; HbA1c = glycated hemoglobin; HDLc = high density lipoprotein cholesterol; LDLc = low density lipoprotein cholesterol; SBP = systolic blood pressure.

**Table S4. Associations of rs671 genotype with outcomes in men and women, adjusted for alcohol drinking**

| Predictor | BMI | | SBP | | DBP | | Log transformed fasting glucose | | Log transformed HbA1c | | Log transformed triglycerides | | HDLc | | LDLc | |
| --- | --- | --- | --- | --- | --- | --- | --- | --- | --- | --- | --- | --- | --- | --- | --- | --- |
|  | **β** | ***P*** | **β** | ***P*** | **β** | ***P*** | **β** | ***P*** | **β** | ***P*** | **β** | ***P*** | **β** | ***P*** | **β** | ***P*** |
| Men (n=46547)  Number of G alleles | | | | | | | | | | | | | | | | |
| 0 | *Ref* |  | *Ref* |  | *Ref* |  | *Ref* |  | *Ref* |  | *Ref* |  | *Ref* |  | *Ref* |  |
| 1 | 0.105 | 0.097 | 0.516 | 0.076 | 0.328 | 0.081 | -0.002 | 0.542 | -0.000 | 1.000 | -0.017 | 0.088 | **0.504** | **0.010** | -0.717 | 0.200 |
| 2 | **0.194** | **0.002** | **0.644** | **0.026** | **0.684** | **<0.001** | -0.003 | 0.289 | -0.004 | 0.096 | 0.007 | 0.506 | **1.226** | **<0.001** | -0.859 | 0.123 |
| Per-allele increase | **0.094** | **<0.001** | **0.240** | **0.048** | **0.348** | **<0.001** | -0.001 | 0.239 | **-0.003** | **0.005** | **0.012** | **0.004** | **0.659** | **<0.001** | -0.308 | 0.188 |
| Women (n=82485)  Number of G alleles | | | | | | | | | | | | | | | | |
| 0 | *Ref* |  | *Ref* |  | *Ref* |  | *Ref* |  | *Ref* |  | *Ref* |  | *Ref* |  | *Ref* |  |
| 1 | -0.042 | 0.415 | **-0.492** | **0.042** | -0.161 | 0.249 | -0.004 | 0.059 | -0.003 | 0.061 | **-0.016** | **0.030** | **0.525** | **0.003** | -0.068 | 0.875 |
| 2 | -0.022 | 0.657 | **-0.754** | **0.002** | -0.183 | 0.184 | **-0.004** | **0.049** | **-0.003** | **0.042** | **-0.024** | **0.001** | **0.606** | **0.001** | -0.237 | 0.577 |
| Per-allele increase | 0.002 | 0.918 | **-0.327** | **0.001** | -0.061 | 0.283 | -0.001 | 0.163 | -0.001 | 0.129 | **-0.010** | **0.001** | **0.206** | **0.005** | -0.141 | 0.422 |

**Abbreviations:**

BMI = body mass index; DBP = diastolic blood pressure; HbA1c = glycated hemoglobin; HDLc = high density lipoprotein cholesterol; LDLc = low density lipoprotein cholesterol; SBP = systolic blood pressure.

**Table S5. Confounder adjusted multivariable and instrumental variable associations of alcohol drinking with cardiovascular disease risk factors, after excluding participants with history of alcohol abuse or cardiovascular disease or stroke**

|  | Mean difference of each outcome comparing current or former drinkers to never or seldom drinkers | | | | | | | | | | | | | | | |
| --- | --- | --- | --- | --- | --- | --- | --- | --- | --- | --- | --- | --- | --- | --- | --- | --- |
|  | **BMI** | | **SBP** | | **DBP** | | **Log transformed fasting glucose** | | **Log transformed HbA1c** | | **Log transformed triglycerides** | | **HDLc** | | **LDLc** | |
|  | **β** | ***P*** | **β** | ***P*** | **β** | ***P*** | **β** | ***P*** | **β** | ***P*** | **β** | ***P*** | **β** | ***P*** | **β** | ***P*** |
|  | **Men (n=41796)** | | | | | | | | | | | | | | | |
| Multivariable analysis^1^ | | | | | | | | | | | | | | | | |
|  | **0.241** | **<0.001** | **3.022** | **<0.001** | **2.485** | **<0.001** | **0.016** | **<0.001** | -0.002 | 0.317 | **0.083** | **<0.001** | **2.546** | **<0.001** | **-4.269** | **<0.001** |
| Instrumental variable analysis^1^ | | | | | | | | | | | | | | | | |
| rs1229984 | 0.507 | 0.735 | **20.418** | **0.004** | 6.826 | 0.130 | -0.092 | 0.190 | -0.099 | 0.064 | -0.031 | 0.897 | 5.673 | 0.220 | 2.712 | 0.838 |
| *P* for difference^2^ | 0.859 | | 0.008 | | 0.330 | | 0.113 | | 0.057 | | 0.633 | | 0.497 | | 0.598 | |
| rs671 | **1.026** | **<0.001** | **5.919** | **<0.001** | **5.259** | **<0.001** | **0.024** | **0.013** | -0.006 | 0.442 | **0.258** | **<0.001** | **6.672** | **<0.001** | **-6.010** | **0.002** |
| *P* for difference^2^ | **<0.001** | | **0.002** | | **<0.001** | | 0.388 | | 0.572 | | **<0.001** | | **<0.001** | | 0.352 | |
|  | **Women (n=73997)** | | | | | | | | | | | | | | | |
| Multivariable analysis | | | | | | | | | | | | | | | | |
|  | **-0.219** | **0.011** | 0.648 | 0.075 | **1.179** | **<0.001** | 0.001 | 0.728 | **-0.014** | **<0.001** | 0.017 | 0.157 | **4.255** | **<0.001** | **-3.104** | **<0.001** |
| Instrumental variable analysis | | | | | | | | | | | | | | | | |
| rs1229984 | 1.501 | 0.729 | -26.511 | 0.163 | **-25.323** | **0.045** | -0.213 | 0.197 | -0.175 | 0.145 | -0.880 | 0.151 | 5.717 | 0.708 | 24.572 | 0.501 |
| *P* for difference | 0.691 | | 0.138 | | **0.023** | | 0.181 | | 0.166 | | 0.128 | | 0.924 | | 0.444 | |
| rs671 | 0.896 | 0.352 | **-15.625** | **<0.001** | -1.631 | 0.528 | -0.056 | 0.115 | **-0.063** | **0.015** | **-0.428** | **0.001** | **11.678** | **0.001** | -11.998 | 0.136 |
| *P* for difference | 0.244 | | **<0.001** | | 0.275 | | 0.106 | | 0.055 | | **<0.001** | | **0.028** | | 0.266 | |

**Abbreviations:**

BMI = body mass index; DBP = diastolic blood pressure; HbA1c = glycated hemoglobin; HDLc = high density lipoprotein cholesterol; LDLc = low density lipoprotein cholesterol; SBP = systolic blood pressure.

^1^ Adjusted for age, education, marital status, smoking and exercise habits.

^2^ Durbin-Wu-Hausman statistic.

**Table S6. Summary of study findings**

| Analysis | BMI | SBP | DBP | Fasting glucose | HbA1c | Triglycerides | HDLc | LDLc |
| --- | --- | --- | --- | --- | --- | --- | --- | --- |
| Men |  |  |  |  |  |  |  |  |
| Association of rs1229984 with outcomes |  |  |  |  |  |  |  |  |
| Association of rs671 with outcomes |  |  |  |  |  |  |  |  |
| Association of rs1229984 with outcomes, adjusted for alcohol drinking |  |  |  |  |  |  |  |  |
| Association of rs671 with outcomes, adjusted for alcohol drinking |  |  |  |  |  |  |  |  |
| Standard multivariable analysis |  |  |  |  |  |  |  |  |
| Standard multivariable analysis (sensitivity analysis) |  |  |  |  |  |  |  |  |
| rs1229984-instrumented analysis |  |  |  |  |  |  |  |  |
| rs1229984-instrumented analysis (sensitivity analysis) |  |  |  |  |  |  |  |  |
| rs671-instrumented analysis |  |  |  |  |  |  |  |  |
| rs671-instrumented analysis (sensitivity analysis) |  |  |  |  |  |  |  |  |
| Women |  |  |  |  |  |  |  |  |
| Association of rs1229984 with outcomes |  |  |  |  |  |  |  |  |
| Association of rs671 with outcomes |  |  |  |  |  |  |  |  |
| Association of rs1229984 with outcomes, adjusted for alcohol drinking |  |  |  |  |  |  |  |  |
| Association of rs671 with outcomes, adjusted for alcohol drinking |  |  |  |  |  |  |  |  |
| Standard multivariable analysis |  |  |  |  |  |  |  |  |
| Standard multivariable analysis (sensitivity analysis) |  |  |  |  |  |  |  |  |
| rs1229984-instrumented analysis |  |  |  |  |  |  |  |  |
| rs1229984-instrumented analysis (sensitivity analysis) |  |  |  |  |  |  |  |  |
| rs671-instrumented analysis |  |  |  |  |  |  |  |  |
| rs671-instrumented analysis (sensitivity analysis) |  |  |  |  |  |  |  |  |

**Abbreviations:**

BMI = body mass index; DBP = diastolic blood pressure; HbA1c = glycated hemoglobin; HDLc = high density lipoprotein cholesterol; LDLc = low density lipoprotein cholesterol; SBP = systolic blood pressure.

**Red cells** indicate detrimental associations and **blue cells** indicate beneficial associations.
